# Aspirin use and risk of depression: a cross-sectional study

**DOI:** 10.3389/fphar.2026.1721286

**Published:** 2026-02-20

**Authors:** Runa A, Huan Yu, Kun Wang, Ruotong Yang, Huairong Wang, Liuyan Zheng, Jingxian Wu, Yiqun Wu

**Affiliations:** 1 School of Public Health, Peking University Health Science Center, Beijing, China; 2 Department of Epidemiology and Biostatistics, School of Public Health, Peking University Health Science Center, Beijing, China; 3 Key Laboratory of Epidemiology of Major Diseases (Peking University), Ministry of Education, Beijing, China

**Keywords:** aspirin, cross-sectional study, depression, NHANES, pharmacoepidemiology

## Abstract

**Background:**

The association between aspirin use and depression risk remains controversial.

**Methods:**

Based on the National Health and Nutrition Examination Survey (NHANES) database from the 2011 to 2018, a cross-sectional study was designed. Depression was estimated using Patient Health Questionnaire-9 (PHQ-9). Multivariable logistic regression models were used to assess the association between aspirin use and depression, adjusting for sociodemographic, lifestyle, and clinical covariates. To explore potential heterogeneity, we stratified the analysis by depression severity and aspirin dose categories.

**Results:**

A total of 4,887 participants with a mean age of 65.5 years were included. Of the participants, 1,421 (29%) were identified to be with depression. Aspirin use was inversely associated with the depression (*OR* = 0.69; 95% *CI*: 0.55, 0.86; *p <* 0.01). For depression of different severity, aspirin use was inversely significantly associated with “Mild depression” (*OR* = 0.65; 95% *CI*: 0.51, 0.83; *p <* 0.001) while not significant associated with “Moderate and severe depression”.

**Conclusion:**

Aspirin use was inversely associated with depression, particularly mild depression, and the association differed by depression severity and aspirin dose. The clinical benefits and risks of aspirin should be carefully considered.

## Highlights

- Aspirin use was inversely associated with the depression, especially with “Mild depression”, but not significantly with “Moderate and severe depression”.
- For different doses of aspirin, significantly inverse association with depression was found both in the low dose and in the high dose.

## 1 Introduction

Depression causes considerable disability and disease burden worldwide, with 332 million prevalent cases in 2021 (World Health Organization, 2025). Though its etiology is not fully understood, inflammation has been indicated to be involved in the pathogenesis of depression (Nestler et al., 2002). Previous studies have shown increased pre-depression levels of inflammatory cytokines such as IL-1β, IL-6, and TNF-α in human (Beurel et al., 2020; Nedic et al., 2021). Due to inflammation, the levels of cortisol and cytokines are elevated (Schiepers et al., 2005), leading to disturbances in tryptophan metabolism and production of 5-hydroxytryptamine (serotonin) and predispose to clinical depression (Konsman et al., 2002). Aspirin, also known as acetylsalicylic acid, is a commonly used nonsteroidal anti-inflammatory drug (NSAID) by reducing pro-inflammatory cytokines. Pharmacological inhibition of astrocytic Mysm1 by aspirin has been shown to alleviate depressive-like behaviors in mice, suggesting its potential role in countering the development of depression (Zhang et al., 2022). However, the relationship between aspirin use and the risk of depression is controversial in population studies. The severity of depression was relieved four and 8 weeks after the adjunctive therapy of aspirin in a clinical trial for major depressive disorder (MDD) patients (Sepehrmanesh et al., 2017), while aspirin use was not associated with reduced risk of depression in several cohort studies (Glaus et al., 2015; Berk et al., 2021; Veronese et al., 2018). In a recent meta-analysis, a higher risk of depression has been reported after aspirin use with an estimated OR value of 1.10 (95% CI: 1.05–1.16) (Kim et al., 2020). The inconsistency observed in previous results may be attributed to variations in the study design, such as the inclusion of depression patients with varying levels of severity and differences in the dosage of aspirin administered to the participants. The relationship between aspirin use and depression with varying severity may be different and remains to be explored. In addition, the dose of aspirin used for depression is also worth exploring, as one cohort study reported a decreased risk of depression after continued use of low-dose aspirin but increased risk after high-dose (Kessing et al., 2019). Another cohort study also suggested the potential of adjunctive antidepressant effects by concomitant use of low-dose acetylsalicylic acid and selective serotonin reuptake inhibitors (Köhler et al., 2015). Therefore, in this study, we aimed to assess the association between aspirin use and the severity of depression, and the association between depression and different doses of aspirin.

### 1.1 Open data, open materials, open code

All data, materials, and code used in this study are available on the following website: https://wwwn.cdc.gov/nchs/nhanes/Default.aspx.

## 2 Methods

### 2.1 Data source and population

A cross-sectional study design was conducted based on the National Health and Nutrition Examination Survey (NHANES) database. NHANES is an ongoing program by the National Center for Health Statistics involving a series of independent, nationally representative cross-sectional surveys, designed to assess the health and nutritional status of adults and children in the US (Statistics, 2018). Using both at-home interviews and examinations performed at a mobile examination center, the database contains a wide range of information (e.g., demographic, socioeconomic, dietary, health-related information, medical and physiological measurements, and so on) from a representative population across the U.S. (Statistics, 2018).

Survey data in the NHANES database from 2011 to 2018 were used in this study. Population with completed information of medication and examination of depressive status were included. The inclusion criteria are as follows: First, with complete information of Patient Health Questionnaire-9 (PHQ-9), which was used to assess the depression status; Second, with complete information of “Preventive Aspirin Use” questionnaire, which was used to assess the aspirin use; Third, individual aged 40 and above, since the “Preventive Aspirin Use” questionnaire was exclusively administered to those over 40. The final analyzed sample consisted of a total of 4,887 individuals. Participants with missing continuous variables were excluded.

### 2.2 Aspirin usage

Data on current aspirin use as well as different doses of aspirin use were ascertained from the “Preventive Aspirin Use” questionnaire, which was administered by trained interviewers to all survey participants 40 years and older between 2011 and 2018. Participants were classified as using aspirin if they reported following their physician’s advice and took aspirin (including sometimes), and not using aspirin if they reported not following their physician’s advice or not tooking aspirin. We further divided aspirin-using participants into low dose (≤100 mg) aspirin use and high dose (>100 mg) aspirin use through the daily aspirin dose, which is calculated by the frequency of taking tablets and the dose per tablet.

### 2.3 Depression assessment

Patient Health Questionnaire-9 (PHQ-9) was used to assess the depression status of participants in the NHANES database, of which the questions were asked at the Mobile Examination Center (MEC) by trained interviewers using the Computer-Assisted Personal Interview (CAPI) system (Statistics, 2018). The PHQ-9 is a reliable and valid tool for the diagnosis of common depressive disorders and related psychiatric disorders (Zimmerman, 2019). It consists of 9 questions, which is scored according to the criteria of the Diagnostic and Statistical Manual of Mental Disorders (DSM), as follows: score 0 for “never”, score 1 for “a few days”, score 2 for “more than a week”, and score 3 for “almost every day”. The total score on the PHQ-9 is calculated by summing the scores for the nine symptom items (range, 0–27). The PHQ-9 is increasingly used as a continuous measure of depression severity. Considering mild depression, depression is defined as a score of 5 or more on the PHQ-9 in the study (Yu et al., 2020; Andrea et al., 2016; Kroenke et al., 2010; Kroenke et al., 2001). We further classified participants into none (0–4), mild (5–9), moderate and severe (10–27) groups (Yu et al., 2020).

### 2.4 Covariates

Several covariates included sex, age, ethnicity, educational category, work status, comorbidity conditions, body mass index (BMI), alcohol consumption status, smoking status, physical activity status, other NSAID use and inflammatory markers were adjusted in the study. Of all the covariates, age and Inflammatory markers were included in the model as a continuous variable and the other covariates were categorical. Race was categorized as non-Hispanic white, non-Hispanic black, Mexican American, other Hispanic, and other races (including multi-racial). Education was categorized as less than high school, high school or equivalent, and college or above. Work status is categorized as working or not working, being looking for a job and not working is defined as not working, and having a job and having a job but not working last week is defined as working. We chose the presence of comorbidities as follows: diabetes mellitus, hypertension, cardiovascular diseases (congestive heart failure, coronary heart diseases, angina, stroke, or heart attack), kidney failure, hepatopathy, cancer, and arthritis, and dividing the number of the comorbidities into 0, 1 and 2 or above (Guirguis-Blake et al., 2022). BMI was categorized as normal weight (BMI ≤25.00 kg/m2), overweight (BMI ≥25.00 kg/m2) and obesity (BMI ≥30.00 kg/m2). Alcohol consumption status was categorized as ''none'', ''mild'' and ''severe'', with “none” defined as not having consumed more than 12 drinks in the past or not having consumed any kind of alcohol, “mild” was defined as ≤1 drink per day for women and ≤2 drinks per day for men, and “severe” was defined as >1 drink per day for women and >2 drinks per day for men (Agriculture UDo, 2020). Smoking status was categorized into “none” and “yes”, with “none” defined as not having smoked more than 100 cigarettes, “yes” defined as having smoked more than 100 cigarettes (Agriculture UDo, 2020). Physical activity was defined as whether or not in a typical week there were “vigorous physical activities” “moderate physical activities” or “none”. Use of other NSAIDs was defined as ever having used any non-aspirin NSAID according to the ATC/TTT classification system, regardless of frequency or duration (ATC/DDD Index, 2026). Inflammatory markers of the WBC count, systemic immune-inflammation index (SII) and systemic inflammation response index (SIRI) were added to the regression analysis (Li et al., 2023). In the sensitivity analysis, additional covariates such as CVD, diabetes, arthritis, and antidepressant medication were considered. All of them were treated as binary variables and the information was obtained from the questionnaire.

### 2.5 Statistical analysis

Due to complex multi-stage probability sampling methodology of NHANES, the sample weights were recalibrated in this study to ensure nationally representative results. Specifically, since NHANES releases a weighted value every 2 years, the sum of these values equals the non-institutionalized civilian population in the United States. The use of standard recalibration ensures that the combined weights accurately represent the average annual population during the period and maintain correct variance estimation. All analyses were conducted using the “survey” tool package in R, and the main sampling unit (SDMVPSU) and stratification variable (SDMVSTRA) were clearly defined to accurately reflect the design of the survey. The recalibrated weights to properly account for the complex survey structure via the “svydesign ()” function (Statistics, 2018). Descriptions of demographic characteristics were firstly performed. Aspirin use, other NSAID use, and depression were then described stratified by sex. We further compared the characteristics of inflammatory markers between participants with depression and those without. Normally distributed continuous variables were showed as mean and standard deviation (SD), differences between groups were compared by t-test. Non-normally distributed continuous variables were performed as median and range interquartile, and differences between groups were compared by Kruskal test. For categorical variables, frequency and percentage values were used for characterization and group differences were tested using chi-square test. Missing data of categorical covariates were recorded as a separate category for the following analysis. The trend test performed a linear fit on both ordinal categorical variables and continuous variables. The resulting P value for the coefficient of this continuous score is reported as the P value for trend. The logistic regression model was established to estimate the associations of aspirin with depression by different aspirin doses and levels of depression severity. Covariates including sex, age, ethnicity, educational category, work status, comorbidity conditions, body mass index (BMI), alcohol consumption status, smoking status, physical activity status, other NSAID use and Inflammatory markers were considered and adjusted in the model. Subgroup analyses were further conducted by age and sex. Several sensitivity analyses were further conducted. First, we estimate associations of aspirin doses and mild depression. Second, we further adjusted the models for CVD, diabetes, arthritis, and antidepressant medication. Third, we tested for interactions between aspirin use and age, sex, CVD, diabetes, and antidepressant medication, and performed stratified analyses by CVD and diabetes status. Fourth, we categorized aspirin dosage into ≤100 mg/day, 100–300 mg/day, and >300 mg/day to better explore the dose–response relationship. Fifth, we conducted subgroup analyses according to tertiles of SII and SIRI, respectively, to further explore the influence of inflammation on the association. Data were analyzed using R Studio 4.2.1. Statistical analyses were performed using two-sided tests, with *p* < 0.05 being considered statistically significant.

## 3 Results

### 3.1 Population characteristic

A total of 4,887 participants with an average age of 65.5 years were included in this study. Table 1 showed demographic characteristics of the participants stratified by sex. Compared with males, females had a higher proportion of other Hispanic, non-working, higher BMI, lower proportion of ever drinking or smoking, and fewer physical activities (*p* < 0.05 for each).

**TABLE 1:** Demographic characteristic of participants.

Characteristics	All participants (n = 4,887)	Male (N = 2,565)	Female (N = 2,322)	*p* value
Age, mean (SD)	65.5 (10.6)	65.3 (10.5)	65.7 (10.8)	0.20
Education level	​	​	​	0.14
Less than high school	1,171 (24%)	592 (23%)	579 (25%)	​
High school	1,186 (24%)	611 (24%)	575 (25%)	​
College	2,526 (52%)	1,361 (53%)	1,165 (50%)	​
Ethnicity	​	​	​	<0.01
Mexican American	523 (11%)	280 (11%)	243 (10%)	​
Other Hispanic	505 (10%)	224 (8.7%)	281 (12%)	​
Non-Hispanic white	2,158 (44%)	1,159 (45%)	999 (43%)	​
Non-Hispanic black	1,187 (24%)	613 (24%)	574 (25%)	​
Other races	514 (11%)	289 (11%)	225 (9.7%)	​
Work	​	​	​	<0.001
None	3,289 (67%)	1,616 (63%)	1,673 (72%)	​
Yes	1,596 (33%)	947 (37%)	649 (28%)	​
BMI	30.4 (6.9)	29.7 (6.0)	31.2 (7.6)	<0.001
Smoking status	​	​	​	<0.001
None	2,340 (48%)	943 (37%)	1,397 (60%)	​
Yes	2,544 (52%)	1,621 (63%)	923 (40%)	​
Drinking status^a^	​	​	​	<0.001
None	698 (14%)	174 (7%)	524 (23%)	​
Yes	1,754 (36%)	847 (33%)	907 (39%)	​
Physical activity	​	​	​	<0.001
None	3,091 (63%)	1497 (58%)	1594 (69%)	​
Yes	1,796 (37%)	1,068 (42%)	728 (31%)	​
Comorbidity	​	​	​	0.12
None	416 (8%)	240 (9%)	176 (7%)	​
One	1,005 (21%)	527 (21%)	478 (21%)	​
Two or above	3,464 (71%)	1,797 (70%)	1,667 (72%)	​

Abbreviation. BMI, indicates body mass index.

^a^ Due to the absence of data, the sum of the percentages does not equal 1.

Table 2 summarized the anti-inflammatory drug use and depression severity of the study population. Aspirin was used by 74% of participants, while other NSAID was consumed by 12% of participants. Based on PHQ-9, a total of 1,421 (29%) cases of depression was diagnosed. Female participants were less likely using aspirin and were more likely using other NSAID, and the female participants were with a higher proportion of depression (*p* < 0.001 for each). Notably, participants younger than 65 years had a higher prevalence of depression, particularly moderate and severe depression (*p* < 0.001).

**TABLE 2:** Aspirin use, other NSAID use, and depression.

Characteristics	All participants (n = 4,887)	Male (N = 2,565)	Female (N = 2,322)	*p* value
Aspirin dose	​	​	​	<0.001
I. None	1,265 (26%)	622 (24%)	643 (28%)	​
II. Yes	3,622 (74%)	1,943 (76%)	1,679 (72%)	​
i. Low dose	3,157 (65%)	1,644 (64%)	1,513 (65%)	​
ii. High dose	465 (10%)	299 (12%)	166 (7%)	​
Other NSAID use	​	​	​	<0.001
None	4,283 (88%)	2,301 (90%)	1,982 (85%)	​
Yes	604 (12%)	264 (10%)	340 (15%)	​
Depression	​	​	​	<0.001
I. None	3,466 (71%)	1,975 (77%)	1,491 (64%)	​
II. With depression	1,421 (29%)	590 (23%)	831 (36%)	​
i. Mild	890 (18%)	377 (15%)	513 (22%)	​
ii. Moderate and severe	531 (11%)	213 (8%)	318 (14%)	​

Abbreviation. NSAIDs, indicates nonsteroidal anti-inflammatory drug.

### 3.2 Inflammatory markers and depression severity

Inflammatory markers in participants with different levels of depression severity were shown in Table 3. Inflammatory markers increased with the increase of depression levels (*P* for trend <0.05 for each). Participants with moderate or severe depression had higher WBC counts, SII and SIRI.

**TABLE 3:** Inflammatory markers and depression severity.

Depression condition	WBC counts Mean (SD)	SII^3^ Mean (SD)	SIRI^3^ Mean (SD)
All participants	7.3 (2.9)	544.1 (390.8)	1.4 (1.1)
I. Non-depression	7.2 (3.0)	532.7 (389.8)	1.4 (1.2)
II. With depression	7.6 (2.4)	572.0 (392.3)	1.5 (1.1)
i. Mild depression	7.5 (2.2)	569.4 (379.3)	1.5 (1.1)
ii. Moderate and severe depression	7.8 (2.8)	576.4 (413.9)	1.5 (1.1)
*P* for trend	<0.001	<0.001	0.036

Abbreviation. WBC, indicates white blood cell; SII, systemic immune-inflammation index; SIRI, systemic inflammation response index.

### 3.3 Associations of aspirin use with different levels of depression

**FIGURE 1:**
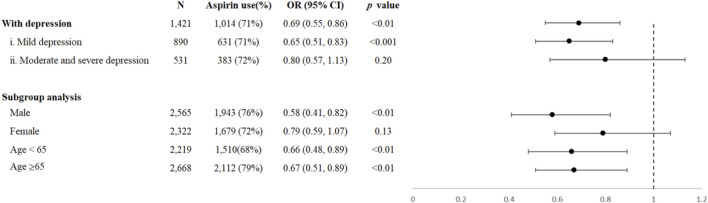
Associations of aspirin use with depression and subgroup analysis. Abbreviation. OR indicates odds ratio.

Figure 1 showed that aspirin use was significantly negatively associated with the depression (*OR* = 0.69; 95% *CI*: 0.55, 0.86; *p <* 0.01), which maintained similar in the subgroup analysis of age and sex (Figure 1). No significant association of aspirin use with depression was found in females (*OR* = 0.79; 95% *CI*: 0.59, 1.07; *p =* 0.013).

Participants with depression were further categorized as mild depression, moderate and severe depression according to PHQ-9. Aspirin use was still significantly negatively associated with mild depression (*OR* = 0.65; 95% *CI*: 0.51, 0.83; *p <* 0.001), while not significant associated with moderate and severe depression (*OR* = 0.80; 95% *CI*: 0.57, 1.13; *p =* 0.20), as shown in the Figure 1.

### 3.4 Associations of different-doses aspirin use with depression

To further explore the associations of aspirin doses with depression, aspirin use was divided into low dose and high dose (Table 4). The results showed that aspirin use was significantly negatively associated with the depression both in low dose (*OR* = 0.70; 95% *CI*: 0.55, 0.88; *p <* 0.01) and in high dose (*OR* = 0.65; 95% *CI*: 0.50, 0.85; *p <* 0.01).

**TABLE 4:** Associations of different-doses aspirin use with depression in subgroups.

Aspirin use	Low dose aspirin use			High dose aspirin use		
	population (n%)	OR	*p*-value	population (n%)	OR	*p*-value
All participants	3,157 (87%)	0.70 (0.55, 0.88)	<0.01	465 (13%)	0.65 (0.50, 0.85)	<0.01
Male	1,644 (85%)	0.62 (0.44, 0.87)	<0.01	299 (15%)	0.43 (0.24, 0.76)	<0.01
Female	1,513 (90%)	0.77 (0.56, 1.06)	0.11	166 (10%)	0.99 (0.61, 1.63)	0.98
Age< 65	1,318 (87%)	0.67 (0.49, 0.93)	0.020	192 (13%)	0.55 (0.35, 0.87)	0.01
Age≥ 65	1,839 (87%)	0.68 (0.51, 0.90)	<0.01	273 (13%)	0.77 (0.47, 1.26)	0.29

Abbreviation. OR, indicates odds ratio. Individuals who did not use aspirin served as the reference group.

Subgroup analyses showed that the negatively association of low dose aspirin with depression was significant in males, participants aged <65 years or ≥65 years, the inversely association of high dose aspirin with depression was significant in males and participants aged <65 years (Table 4). Subgroup analysis by race/ethnicity is presented in Supplementary Table S4. Among Non-Hispanic White participants, we observed an inverse association between aspirin use and depression (*OR* = 0.62; 95% *CI*: 0.46, 0.82; *p <* 0.01).

Further sensitivity analyses were conducted to estimate the associations of aspirin use with mild depression. As shown in Supplementary Table S2, low-dose aspirin use was associated with mild depression for all the subgroups (*p* < 0.05 for each), while the negatively association of high dose aspirin use with mild depression was significant only in males (*p* = 0.046). As shown in Table 3, aspirin users had a higher prevalence of CVD (*p* < 0.001) or diabetes (*p* < 0.01). After further adjusting for CVD, diabetes, arthritis, and antidepressants, our primary finding remained robust: low-dose aspirin was associated with significantly lower odds of depression (OR = 0.67; 95% CI: 0.54, 0.84; *p* < 0.001), and the association was even stronger for high-dose aspirin (OR = 0.61; 95% CI: 0.46, 0.82; p < 0.01). No significant interactions were observed between aspirin use and age, sex, CVD, diabetes, or antidepressant medication. (p _interaction_ > 0.05 for each) (Supplementary Table S5). More refined stratification by aspirin dose, as well as stratification by inflammation, both support our main findings (Supplementary Tables S6, S7).

## 4 Discussion

With a cross-sectional design using NHANES database from 2011 to 2018 which was representative nationally, this study found that aspirin use was significantly negatively associated with the depression.

We found heterogeneity in the associations of aspirin use with different levels of depression. In this study, aspirin use was significantly inversely associated with mild depression (PHQ-9 scores 5–9), but not with moderate or severe depression (PHQ-9 ≥10). This difference in statistical significance was likely driven in part by smaller sample sizes in the higher-severity groups. Moreover, individuals with PHQ-9 scores of 5–9 and those with scores ≥10 exhibited distinct clinical characteristics, behavioral traits, and treatment patterns (Kroenke et al., 2001), which may underlie the observed differences in association. Our findings are supported by some prior evidence (Sepehrmanesh et al., 2017; Köhler et al., 2015). However, they differ from the results reported in the 2020 review by Kim et al. (Kim et al., 2020), primarily because that review included highly heterogeneous populations—mostly from Europe—with considerable variation across studies in design, lack of distinction by aspirin dose, and vastly different definitions of depression. Another study found that high-dose aspirin use (>500 mg/day) was associated with an increased risk of depression (Kessing et al., 2019); In contrast, in our study, high-dose aspirin was defined as >100 mg/day, and virtually no participants used doses exceeding 500 mg/day.

Importantly, our decision to analyze mild depression as a separate category was deliberate and grounded in both clinical relevance and methodological precedent. First, this categorization aligns with widely adopted approaches in the literature; numerous prior studies have used similar PHQ-9 groupings to define subthreshold or mild depressive symptoms (Yu et al., 2020; Andrea et al., 2016; Kroenke et al., 2010; Kroenke et al., 2001). Notably, a PHQ-9 cutoff of ≥5 demonstrates high sensitivity for detecting clinically relevant depression (Kroenke et al., 2001). Second, accumulating evidence indicates that even mild or subclinical depression is significantly associated with functional impairment, elevated risk of chronic diseases, and increased likelihood of progression to more severe depression (Scodari et al., 2023) — underscoring its importance as a target for early intervention. Given that one key objective of our study was to examine the association between aspirin use and depression across varying levels of symptom severity, distinguishing the 5–9 group allowed us to capture potential dose–response or threshold effects that might be masked in broader classifications. This disparity in associations across depression severity levels should be noted in clinical practice and future research. The findings of this study suggest that future related RCTs might be better targeted toward mild depression.

Although we observed a significant inverse association between aspirin use and mild depression, this association did not reach statistical significance for moderate and severe depression. While limited statistical power due to the relatively low prevalence of moderate and severe cases in our sample may partly account for this null finding (power = 54% in *post hoc* analysis), biological differences in the pathophysiology of depression severity could also play a role. Mild depression is more strongly linked to subclinical inflammation and vascular dysfunction—pathways that are directly modulated by aspirin’s anti-inflammatory and antiplatelet effects (Janssen et al., 2021). In contrast, moderate and severe depression often involves more complex neurobiological alterations, including HPA axis dysregulation, reduced neuroplasticity, and monoaminergic dysfunction, which may be less responsive to aspirin’s primary mechanisms of action (Konsman et al., 2002; Kroenke et al., 2001).

In this study, both low and high doses of aspirin were significantly inversely associated with depression. Although previous studies have suggested that low-dose aspirin reduced the risk of depression, the relationship between high-dose aspirin and depression was still controversial. There was a study suggesting that high-dose aspirin is associated with an increased incidence of depression (Kessing et al., 2019), whereas another study suggesting that co-administration of N-Acetylcysteine and 1000 mg aspirin over 16 weeks is associated with reductions in bipolar depressive symptoms (Bauer et al., 2018). Different doses of aspirin use on depression need to be further explored.

The subgroup results of our study showed both low dose and high dose aspirin reversely associated with depression, while low dose aspirin use on depression was more significant at age ≥65 years, and the high dose were more significant at age <65 years. As shown in Supplementary Table S2, a higher proportion of mild depression were estimated in participants with depression over 65 years, while a higher proportion of moderate and severe depression were found in participants with depression below 65 years. One possible explanation is dose-response relationship and optimal dose. The existence of studies showed no significant associations of aspirin with depression in participants aged <66 years and at an average 37 years (Glaus et al., 2015; Molero et al., 2019), and another study showed no significant effect of aspirin in 15–25 years participants with 100 mg aspirin (Berk et al., 2020), suggesting that the associations of low- and high-dose aspirin with depression vary by age group. Moreover, it is noteworthy that high-dose aspirin is rarely used in clinical practice for cardiovascular disease prevention; instead, it is more commonly prescribed for the treatment of pain or inflammatory conditions. This difference in indications between low- and high-dose aspirin may introduce potential confounding. In the present study, we adjusted for both NSAID use and inflammatory markers, yet the inverse association between high-dose aspirin and depression risk persisted. The biological plausibility for this finding may lie in aspirin’s dose-dependent pharmacological effects: its stronger inhibition of cyclooxygenase-2 at higher doses could lead to greater suppression of pro-inflammatory cytokine, which have been implicated in the pathogenesis of depression. This anti-inflammatory action could theoretically contribute to mood improvement, particularly among individuals with elevated inflammatory burden.

While our findings suggest a potential inverse association between aspirin use and depression, the clinical applicability of aspirin in older adults must be carefully weighed against its safety profile, especially in those with a high comorbidity burden. Aspirin, especially high-dose aspirin, is associated with an increased risk of gastrointestinal bleeding, renal impairment, and hemorrhagic stroke—complications that are markedly amplified in older individuals with multimorbidity, polypharmacy, or frailty (McNeil et al., 2018). Indeed, guidelines for cardiovascular prevention in older adults increasingly recommend against initiating aspirin for primary prevention in individuals aged ≥60 years and suggest discontinuation in those who have already started treatment at age 75 years considering increasing bleeding risk (Davidson et al., 2022). Therefore, if indicated at all, should align with established indications and be prescribed at the lowest effective dose, with careful assessment of bleeding risk, renal function, and concomitant medications. Future research should explore safer anti-inflammatory strategies for depression in this vulnerable group.

The subgroup results of our study showed that the association between aspirin use and depression was statistically significant in males but not in females, although the interaction between sex and aspirin use was not statistically significant. However, some previous evidence suggests that there might be potential differences between men and women. The potential causes include the widened differences in immune cell profiles between the sexes in older adults (Huang et al., 2021), higher usage rates of other NSAIDs among women, or sex-specific lifestyle factors. Nevertheless, these factors were further controlled in our study, and our results still indicate an inverse association between aspirin use and depression in both men and women—although the association did not reach statistical significance in women. We also conducted a further exploratory analysis in female patients who did not use any other NSAIDs, and found that the association between aspirin use and a lower risk of depression was marginally significant (*OR* = 0.74; 95% *CI*: 0.53, 1.02; *p =* 0.069). This aligns with prior work indicating a protective effect of aspirin against depression in women (Pasco et al., 2010). The interaction between estrogen and aspirin may partly explain this sex difference. Estrogen possesses anti-inflammatory properties and modulates COX activity, which could theoretically synergize with aspirin’s inhibition of pro-inflammatory pathways (Huang et al., 2021; Pasco et al., 2010). However, in our study, the association in women did not reach statistical significance—potentially due to limited power, or heterogeneity in menopausal status. In men, findings are heterogeneous. A study including 5,556 men aged 69–87 years in Australia suggests that aspirin use may increase the risk of depression in later life (Almeida et al., 2010), on the contrary, a further distinction was made between men aged 69–87 years with high levels of total plasma homocysteine (N = 3,687) suggests that aspirin reduces the risk of depression (Almeida et al., 2012). In the case of inconsistent evidence, sex subgroup analysis related to the associations of aspirin use with depression needs to be further explored.

To validate our findings, we also conducted additional exploratory analyses. We found that our estimates of the association remained robust after adjusting for the presence or absence of a recent cold and the antidepressant medication. This suggests that recent infection and depression treatment did not cause the misestimation of the associations. In addition, we further explored the clinical diagnosis with ICD-10 code of depression as the study outcome, and did not obtain statistically significant results due to the small number of cases (N = 386) and low study power.

Furthermore, even after controlling for CVD, diabetes, arthritis, and antidepressants, the use of aspirin was still associated with a lower risk of depression in sensitivity analyses. These findings were consistent among subgroups, indicating the stability of our results. This finding carries important clinical implications, further strengthening confidence in the use of aspirin among patients at high cardiovascular risk.

This study has several limitations. First, the cross-sectional design precludes the establishment of temporality, and reverse causality remains plausible, as greater depression severity might lead to reduced aspirin adherence. Evidence from further cohort studies is needed. Second, the PHQ-9 captures depressive symptoms over the past 2 weeks, whereas aspirin use in our study reflects habitual or current intake (typically assessed as use within the past 30 days in NHANES). This discrepancy in time frames could, in principle, introduce misclassification if aspirin use or depressive symptoms were highly variable over short periods. This temporal mismatch limits our ability to establish temporality or causality. Other limitations include the accuracy of recall that may affect self-reported medication use, the lack of aspirin use duration, the lack of homocysteine data not allowing for the subgroup analysis despite established depression links (Almeida et al., 2012), and the inability to investigate the duration of depression.

The burden of disability due to depression is also increasing (Jorm et al., 2017), suggesting high costs associated with depression management from both epidemiological and public policy perspectives. Meanwhile, the many off-target outcomes of aspirin use (Guirguis-Blake et al., 2022), particularly those related to bleeding, may challenge the broad indications of aspirin for primary prevention in older population (McNeil et al., 2018). Aspirin should not be repurposed for depression prevention given bleeding risks (McNeil et al., 2018) in older adults with potential bleeding risk.

Our study shows that aspirin use is inversely associated with depression, and this association may differ by depression severity and aspirin dose. The clinical benefits and risks of aspirin should be carefully considered.

## Data Availability

Publicly available datasets were analyzed in this study. This data can be found here: https://wwwn.cdc.gov/nchs/nhanes/Default.aspx.
