# Supplementary material for: Aspirin use and risk of depression: a cross-sectional study
Source: Front Pharmacol. 2026 Feb 20;17:1721286. doi: 10.3389/fphar.2026.1721286 (PMC12963233; doi:10.3389/fphar.2026.1721286)
Supplement: Supplementary file 1 [file Table1.docx]

**Supplementary Material**

**Supplementary Table 1. Age category and depression severity.**

| **Depression severity (N = 4,887)** | **age< 65 (N = 2,219)** | **age≥65 (N = 2,668)** | **p-value** |
| --- | --- | --- | --- |
| **I. None (N = 3,466)** | 1,481 (67%) | 1,985 (74%) | <0.001 |
| **II. With depression (N = 1,421)** | 738 (33%) | 683 (26%) |  |
| i. Mild (N = 890) | 421 (19%) | 469 (18%) |  |
| ii. Moderate and severe (N = 309) | 317 (14%) | 214 (8.0%) |  |

**Supplementary Table 2. Associations of different-doses aspirin use with mild depression in subgroup analyses.**

|  | **Low dose aspirin use** | | |  | **High dose aspirin use** | | |
| --- | --- | --- | --- | --- | --- | --- | --- |
|  | **population(n%)** | **OR (95% CI)** | **p-value** |  | **population(n%)** | **OR (95% CI)** | **p-value** |
| **All participants (n = 890)** | 554 (62%) | 0.65 (0.5, 0.85) | <0.01 |  | 77 (8.7%) | 0.66 (0.47, 0.92) | 0.016 |
| **Male (n = 377)** | 237 (63%) | 0.63 (0.4, 0.99) | 0.045 |  | 36 (10%) | 0.50 (0.26, 0.99) | 0.046 |
| **Female (n = 513)** | 317 (62%) | 0.67 (0.47, 0.95) | 0.030 |  | 41 (8%) | 0.89 (0.52, 1.54) | 0.68 |
| **Age< 65 (n = 421)** | 234 (56%) | 0.6 (0.41, 0.89) | 0.010 |  | 35 (8%) | 0.58 (0.33, 1.04) | 0.065 |
| **Age≥ 65 (n = 469)** | 320 (68%) | 0.69 (0.5, 0.95) | 0.020 |  | 42 (9%) | 0.74 (0.41, 1.34) | 0.32 |

Abbreviation. OR indicates odds ratio; CI, confidence interval. Individuals who did not use aspirin served as the reference group.

**Supplementary Table 3. Supplementary characteristics of participants by aspirin use status.**

|  | **Aspirin Users** | **Aspirin Non-Users** | ***P*** |
| --- | --- | --- | --- |
| **≥65 years-old** | 52.9% | 41.6% | <0.001 |
| **CVD** | 30.7% | 17.1% | <0.001 |
| **Diabetes** | 29.6% | 23.8% | <0.01 |
| **Arthritis** | 50.4% | 49.1% | 0.57 |

Abbreviation. CVD, cardiovascular disease.

**Supplementary Table 4. Subgroup analyses—association between aspirin and depression stratified by race/ethnicity.**

|  | **N** | **OR (95% CI)** | ***P*** |
| --- | --- | --- | --- |
| **Race** |  |  |  |
| Mexican American | 523 | 0.68 (0.37, 1.23) | 0.18 |
| Other Hispanic | 505 | 0.64 (0.37, 1.10) | 0.10 |
| Non-Hispanic White | 2,158 | 0.62 (0.46, 0.82) | <0.01 |
| Non-Hispanic Black | 1,187 | 1.11 (0.78, 1.58) | 0.56 |
| Other Race | 514 | 0.84 (0.42, 1.67) | 0.61 |

Abbreviation. OR indicates odds ratio; CI, confidence interval.

**Supplementary Table 5. Subgroup analyses and interaction tests for the association between aspirin use and depression risk.**

|  | **OR (95% CI)** | ***P*** | ***P*_interaction_** |
| --- | --- | --- | --- |
| **Age** |  |  | 0.89 |
| <65 years-old | 0.66 (0.48, 0.89) | <0.01 |  |
| ≥65 years-old | 0.67 (0.51, 0.89) | <0.01 |  |
| Interaction | 1.03 (0.68, 1.55) | 0.89 |  |
| **Sex** |  |  | 0.14 |
| Male | 0.58 (0.41, 0.82) | <0.01 |  |
| Female | 0.79 (0.59, 1.07) | 0.13 |  |
| Interaction | 1.42 (0.89, 2.25) | 0.14 |  |
| **CVD** |  |  | 0.83 |
| Yes | 0.54 (0.21, 1.43) | 0.18 |  |
| No | 0.63 (0.48, 0.83) | <0.01 |  |
| Interaction | 1.06 (0.65, 1.72) | 0.83 |  |
| **Diabetes** |  |  | 0.59 |
| Yes | 0.47 (0.25, 0.87) | 0.020 |  |
| No | 0.74 (0.55, 0.99) | 0.040 |  |
| Interaction | 0.90 (0.61, 1.33) | 0.59 |  |
| **Antidepressants** |  |  | 0.51 |
| Yes | 0.78 (0.47, 1.31) | 0.34 |  |
| No | 0.63 (0.49, 0.80) | <0.001 |  |
| Interaction | 1.21 (0.68, 2.15) | 0.51 |  |

Abbreviation: OR indicates odds ratio; CI, confidence interval; CVD, cardiovascular disease. Interaction refers to the product term formed by the use of aspirin and the corresponding covariates.

**Supplementary Table 6. Sensitivity analysis—Association between different doses of aspirin and the risk of depression.**

|  | **OR (95% CI)** | ***P*** |
| --- | --- | --- |
| **Aspirin dose** |  |  |
| ≤100 mg/d | 0.69 (0.55, 0.87) | <0.01 |
| 100-300 mg/d | 0.67 (0.20, 2.24) | 0.51 |
| >300 mg/d | 0.57 (0.43, 0.80) | <0.01 |

Abbreviation: OR indicates odds ratio; CI, confidence interval.

**Supplementary Table 7. Sensitivity analysis—association between aspirin and depression stratified by inflammatory markers.**

|  | **Low dose aspirin** | |  | **High dose aspirin** | |
| --- | --- | --- | --- | --- | --- |
|  | **OR (95% CI)** | ***P*** |  | **OR (95% CI)** | ***P*** |
| **SII** |  |  |  |  |  |
| Low | 0.62 (0.43, 0.89) | 0.011 |  | 0.44 (0.23, 0.86) | 0.016 |
| Middle | 0.75 (0.52, 1.07) | 0.11 |  | 0.77 (0.42, 1.42) | 0.39 |
| High | 0.70 (0.45, 1.10) | 0.12 |  | 0.68 (0.38, 1.20) | 0.17 |
| **SIRI** |  |  |  |  |  |
| Low | 0.55 (0.35, 0.86) | 0.010 |  | 0.38 (0.18, 0.80) | 0.011 |
| Middle | 1.08 (0.68, 1.71) | 0.74 |  | 0.91 (0.47, 1.78) | 0.79 |
| High | 0.53 (0.39, 0.72) | <0.001 |  | 0.54 (0.31, 0.94) | 0.029 |

Abbreviation. Abbreviation: OR indicates odds ratio; CI, confidence interval; SII, systemic immune-inflammation index; SIRI, systemic inflammation response index.
